# Human plasma protein corona decreases the toxicity of pillar-layer metal organic framework

**DOI:** 10.1038/s41598-020-71170-z

**Published:** 2020-09-03

**Authors:** Samira Jafari, Zhila Izadi, Loghman Alaei, Mehdi Jaymand, Hadi Samadian, Vali ollah Kashani, Hossein Derakhshankhah, Payam Hayati, Foad Noori, Kamran Mansouri, Faezeh Moakedi, Jan Janczak, Mohammad Jaafar Soltanian Fard, Nozar Fayaz bakhsh

**Affiliations:** 1 Pharmaceutical Sciences Research Center, Health Institute, Kermanshah University of Medical Sciences, Kermanshah, Iran; 2 Department of Biology and Biotechnology, Faculty of Sciences, University of Kurdistan, Sanandaj, Iran; 3 Nano Drug Delivery Research Center, Health Technology Institute, Kermanshah University of Medical Sciences, Kermanshah, Iran; 4 Department of Physical Education, Faculty of Human Sciences, Semnan University, Semnan, Iran; 5 Persian Gulf Science and Technology Park, Nano Gostaran Navabegh Fardaye Dashtestan Company, Borazjan, Iran; 6 Student’s Research Committee, Faculty of Pharmacy, Kermanshah University of Medical Sciences, Kermanshah, 6714415153 Iran; 7 Medical Biology Research Center, Health Technology Institute, Kermanshah University of Medical Sciences, Kermanshah, Iran; 8 Department of Biochemistry and Molecular Biology, School of Medicine, West Virginia University, Morgantown, USA; 9 Institute of Low Temperature and Structure Research Polish Academy of Sciences, P.O. Box 1410, Okolna 2 str., 50-950 Wrocław, Poland; 10 Department of Chemistry, Faculty of Chemical Science, Firoozabad Branch, Islamic Azad University, P.O. Box 74715-117, Firoozabad, Fars Iran

**Keywords:** Materials science, Biomaterials, Biomaterials - proteins

## Abstract

This scenario was designed to investigate the protein corona pattern on the pillar-layer surface of a Cu-based metal–organic framework (MOF) in human plasma. The [Cu(L)(L^/^)].1.3DMA (MOF-1) {L = 4, 4^/^-bipyridine and L^/^ = 5-aminoisophthalic acid}, was synthesized through the sonochemical irradiation approach as well as characterized by various techniques like scanning electron microscopy, Fourier-transform infrared spectroscopy, X-ray powder diffraction and single-crystal X-ray diffraction. The space group was determined to be an orthorhombic space group (*Pbam*) by single-crystal X-ray diffraction. Single-crystal X-ray analyses on MOF-1 showed that Cu^+2^ ion was 6-coordinated. Besides, to study and clarify interactions between MOFs and biological milieu, human whole blood plasma was selected as a model. Fluorescence spectroscopy and SDS-PAGE techniques were employed to explore quantitative and qualitative in situ characterization of protein corona as well. Furthermore, cell viability in a cancerous cell lines was evaluated by MTT assay in the presence and absence of the corona. The results from SDS-PAGE illustrated that the most adsorbed quantity among plasma proteins belongs to fibrinogen (α, β and γ chains), and this protein showed the maximum frequency on the MOF-1s surface, so the possible interactions of MOF-1s with fibrinogen also studied using fluorescence spectroscopy and corresponding data were plotted. According to the obtained data from MTT assay, these structures have concentration-dependent toxicity. In brief, based on the obtained data in the current study, the designed MOF can be introduced as a new desirable carrier for drug/gen delivery after further prerequisite assessments.

## Introduction

Identification of the proteins that surround nanoparticles may have remarkable effects on the clearance, biodistribution, and toxicity of them. The role of protein-nanoparticle interactions in nanomedicine has begun to the development of the term nanoparticle-protein corona which is classified into two types based on protein affinity toward the NP surface: (a) high affinity proteins which binds tightly to NPs (hard corona) and (b) low affinity proteins whose adsorption is dynamic, and freely exchange during the time^1–4^.

Metal–organic frameworks (MOFs), are porous coordinated networks comprised of metal cations/clusters, the desired group of porous crystalline with exceptional features including tunable structure, high porosity, ultrahigh surface area structures and so on^5^. Owing to their design possibility, MOFs have been extensively employed in the versatile areas from chemical applications (e.g. gas storage and sensors) to biomedical applications (e.g. drug delivery systems and biosensors). According to literature review, various pathways have been reported for the synthesis of these porous materials like layer-by-layer growth, microwave, electrochemical, mechanochemical and sonochemical techniques^6^. Time, product yield as well as cost are the most crucial requirements for MOFs synthesis. Therefore, fulfilling these requirements is possible through the selection of an appropriate strategy for their synthesis. In comparison with the other methods, ultrasound irradiation is an efficient and attractive strategy due to shorter reaction time, high product yield as well as controllable crystallite size^7^.

Owing to extensive applications of MOFs in the context of nanomedicine, investigation of their bio-fate upon their administration pathway in the human body is a crucial issue. The bio-distribution of nanostructures can be affected by various parameters. In this regard, the surface chemistry of nanoparticles possesses a key role in their stability as well as their interactions with cell membranes^8–10^.

It is well documented that once nanoparticles appear in biological fluids such as plasma and cerebrospinal fluid, layers of proteins coat the surface of nanoparticles; these attached proteins are known as the protein corona^11^. These interactions will determine the pharmacological fate of the corona-covered nanoparticle as well as will designate the toxicological profile^12^. The composition of the protein corona plays a significant role in the biological identity of nanostructures, which relates to the synthetic identity and also the physiological environment^13,14^.

In the current study, MOF-1s were synthesized via the sonication technique and their physicochemical properties were evaluated. Then, to distinguish MOF-1s behaviors in biological conditions, body condition was simulated by incubation of MOF-1s in whole plasma media. To explore protein affinity and thermodynamics interactions between MOF-1s and plasma proteins, various analyses were conducted like quantitative and qualitative in situ characterization of the protein-nanoparticle interactions using fluorescence spectroscopy and gel-electrophoresis technique, respectively. Cytotoxicity of the synthesized bare MOF-1s and corona covered MOF-1s were assessed by MTT assay in cancerous cell lines as well. Overall, based on obtained results in the current study, fabricated MOF-1s are potentially suggested as a desirable candidate for drug delivery after further prerequisite assessments.

## Materials and methods

### Materials

All chemicals were of analytical grade and used as received. Healthy human plasma was obtained from the blood transfusion center and pooled for the next step of the experiments.

### Synthesis of MOF-1 as single crystal structure

Solvothermal synthesis has been used to synthesis MOF-1, 4, 4^/^-bipyridine {L} (156 mg, 1 mmol) accompanied with 5-aminoisophthalic acid {L^/^} (181 mg, 1 mmol) were dissolved in 15 ml distilled DMF in the vigorous stirring conditions under ambient temperature for 30 min. In the following, a solution of Cu (NO_3_)_2_·6H_2_O (295 mg, 2 mmol) was slowly added to the prepared solution in the previous step. Then, the reaction mixture was located into a Parr-Teflon lined stainless steel vessel and subsequently, it was sealed and heated to 130 °C for 3 days. Through slow cooling, the produced mixture was cooled by room temperature. Ultimately, the resulted blue crystals of MOF-1 from the final reaction mixture via filtration and air-dried at the ambient temperature, were assessed using single-crystal X-ray diffraction analysis. Analytical calculations for C_23.2_H_24.7_CuN_4.3_O_5.3_: C, 54.42; H, 4.86; N, 11.76. Anal. Found: C, 54.38; H, 4.61; N, 11.53%. IR (selected bands for compound MOF-1; in cm^−1^): 3,307 (s), 3,092 (w), 1,710 (w), 1618 (w), 1581 (w), 1,382 (s)^15^.

### Synthesis of MOF-1 under ultrasonic irradiation

The sonochemical irradiation approach was employed to synthesize the MOF-1. Following immersing a high-density ultrasonic probe into Cu (NO_3_)_2_·6H_2_O (10 mL, 0.1 M), either 4,4′-Bipyridine {L} (10 mL, 0.1 M) and 5-aminoisophthalic acid {L^/^} (10 mL, 0.1 M) were dropwisely added. To examine the effects of various parameters, the process was multiple repeated by changing the value of one parameter each time, as seen the Table 1. In the procedures, the synthesized precipitates were filtered, afterward washed with DMF and dried. Analytical calculations for C_23.2_H_24.7_CuN_4.3_O_5.3_: C, 54.42; H, 4.86; N, 11.76. Anal. Found: C, 54.40; H, 4.62; N, 11.61%. IR (selected bands for MOF-1s; in cm^−1^): 3,324(s), 3,085(w), 1,730(w), 1,640(w), 1535(w), 1,395(s).

**Table 1:** Crystal data and structure refinement for MOF-1.

Empirical formula	C_23.2_H_24.7_CuN_4.3_O_5.3_
Formula weigh	957.88
Temperature	293(2)K
Crystal system	Orthorhombic
Space group	*Pbam*
Unit cell dimensions	a = 14.3040(16) Å, α = 90° b = b = 16.970(2) Å, β = 90° c = 11.098(2) Å, γ = 90°
Volume	2,693.9(7) Å^3^
Z	4
Crystal size	0.27 × 0.23 × 0.19
Density(calculated)	1.263 g/cm^3^
F(000)	1,062
Theta(max)	29.5°
Goodness- of- fit on F^2^	1.105
Refinement	R[F^2^ > 2σ(F^2^)] = 0.148 wR(F^2^) = 0.301
Reflns collected/unique	12,881/2,462
Largest diff. peak and hole	1.90, − 0.87 eA^−3^

### Evaluation of physicochemical properties

As mentioned previously, MOF-1 was synthesized through the sonochemical irradiation approach and subsequently was characterized, which were summarized in the next paragraphs.

The composition of MOF-1 was evaluated using Heraeus CHN-S-Rapid element analyzer. Bruker Tensor 27 FT-IR spectrometer was employed to record the IR spectrum in the range of 4,000–400 cm^−1^ (KBr pellets technique)^16^. The crystalline structure of the MOF-1 was characterized by Oxford Diffraction four-circle diffractometer Gemini A Ultra with Atlas CCD detector using graphite monochromated MoKα radiation (λ = 0.71073 Å) at room temperature^17^. Cell refinement and data reduction were conducted with the CrysAlisPro software package^17^. The structure of MOF-1 was solved by the direct methods using SHELXS and refined by full-matrix least-squares on *F*^2^ by SHELXL-2014. All the non-hydrogen atoms were refined anisotropically, and hydrogen atoms were placed in the calculated positions and refined with riding constrains with *d*(C–H) = 0.93 Å and *U*_iso_(H) = 1.2 *U*_eq_(C). Details of the crystallographic data collection, structural determination, and refinement are described in Table 1. The selected bond lengths and angles are listed in Table 2, as well.

**Table 2:** Selected bond lengths (Å) for MOF-1.

Cu_1_–O_1_	1.993(8)	H_1_–C_3_	0.930(6)
Cu_1_–O_1_	1.251(8)	H_3_–C_7_	0.930(6)
O_4_–C_8_	2.010(5)	Cu_2_–O_2_	2.349(9)
H_8_–N_2_	0.860(5)	C_13_–C_10_	1.371
Cu_1_–O_3_	2.045(6)	C_13_–C_12_	1.370
Cu_2_–O_4_	2.510(6)	N_1_–C_10_	1.314
H_9_–N_2_	0.860(6)	N_1_–Cu_1_	2.010(5)

X-ray powder diffraction (XRPD) measurements were accomplished on a X’pert diffractometer made by Philips with a monochromated CuKα rays. Simulated PXRD patterns were generated from single-crystal X-ray data using the Mercury software. The morphology of the nanostructured samples of MOF-1 was investigated by SEM (S-4200, Hitachi, Japan). The samples were made by deposition of the drop of the materials recently scattered in appropriate solvents on aluminum stubs accompanied with evaporation of the solvent^18^.

Ultrasonic synthesis was conducted through a multiwave ultrasonic generator (Sonicator-3000, Misonix Inc., Farmingdale, NY, USA) equipped with a converter/transducer and titanium oscillator (horn) with 12.5-mm diameter. The generator was operated at 40 kHz with a maximum power output of 600 W at room temperature for 1 h^19^.

Melting point values were measured by utilizing an electrothermal 9,100 device and were uncorrected^20^.

### Hard corona formation

Protein’s natively folded conformation together with its net surface charge, shows the main role in nanoparticle-protein corona formation. Several inter and intra-molecular forces are involved in the interaction at the Nano-bio interface like hydrogen bonds, solvation forces, van der Waals interactions and so on*.* Specific association and dissociation rates for each protein decide the longevity of their interaction with the NP surface. Long-term adsorption of proteins on the NPs surface leads to the formation of a hard corona whereas short-term binding of proteins with faster exchange rates is defined as soft corona^3,21^. Hard corona formation was prepared by incubation of MOF-1s (final concentration of MOF-1s was 100 μg/mL) in 10% and 100% human plasma at 37 °C for 1 h. After that, the solution was centrifuged at 14,000 rpm for 20 min^22^.

The supernatant was discarded, and the pellet was dissolved in phosphate buffer solution (PBS) and centrifuged at 18,000 rpm for 15 min. the washing process was repeated three times. Finally, the hard corona coated-MOF-1s were dissolved in 500 μL PBS and used for next analysis. In order to have a better understanding of the protein composition on the surface of MOF-1s and resulted corona, we studied the corona formation qualitatively by SDS-PAGE technique.

### One-dimensional SDS-PAGE

Gel electrophoresis technique was used to analyze the protein adsorbed on the surface of NPs in qualitatively^23^. In one dimensional SDS-PAGE (1D-SDS-PAGE) proteins are separated based on molecular weight. In this study, MOF-1s coatings were subjected to two different plasma types (10 and 100%). At first, the hard corona-nanoparticle complex obtained from different concentrations of human plasma was solved in the loading buffer and incubated for 10 min at 100 °C temperature. Then, the same amounts from the obtained complex of different concentrations of human plasma were loaded in SDS-PAGE and were analyzed. The mesh of the gel was 15%.

### Cell culture and cytotoxicity assays

The possible cytotoxicity effects of the drug formulations as well as synthesized materials were assessed using MTT assay^24^. For this purpose, in this study, the MCF-7 cell line was maintained in DMEM-high glucose medium supplemented with 10% of fetal bovine serum (FBS) and 1% of penicillin–streptomycin solution. The cytotoxic activity of the MOF-1s on MCF-7 cell line was analyzed by the MTT assay. To this end, 24 h prior to the assay, the cells were seeded in 96-well plates at a density of $2 \times 10^{4}$ cells per well. Then, after the incubation time, cells were treated with different concentrations of MOF-1s (2–32 µg/ml), MOF-1s-plasma 10%, MOF-1s-plasma 100% and phosphate buffered saline (as the control) were prepared and then added to each well and plate kept 24 h at 37 °C with a 5% CO2 atmosphere in incubator. The cytotoxicity was done based on the MTT protocol. The amount of 10 µl of MTT (5 mg/ml) was added to each well and incubated for 4 h at 37 °C until intracellular purple formazan crystals became visible under the microscope. Subsequently, the culture medium was removed and added 100 µl of DMSO to each well and incubated at room temperature in dark for 1–2 h, until cells have lysed and purple crystals have dissolved. The absorbance of cells has been measured microplate reader scanning spectrophotometer (Microplate reader, BioTek Synergy H1, USA) at 570 nm. Relative Cell viability was calculated using the equation below:

$$\% Relative\;cell\;viability = \left( {\frac{{Abs_{sample} - Abs_{blank} }}{{Abs_{control} - Abs_{blank} }}} \right) \times 100{ }$$

where Abs_sample_ is the absorbance of the cells incubated with MOFs suspensions and Abs_control_ is the absorbance of the cells incubated with PBS and wells treated with MTT without the presence of any cell as a blank.

### Fluorescence spectroscopy

Intrinsic fluorescence quenching in exposure to MOFs was recorded on a Hitachi spectrofluorimeter, MPF-4 model, equipped with a thermostatically controlled cuvette compartment. Excitation was performed at 280 nm. Fluorescence emission was measured at different temperatures (25, 37 and 42 °C) in phosphate buffer saline (PBS) containing various concentrations of MOF nanoparticles. The protein concentration was fixed at 0.58 µM. The nanoparticle concentration range was from 8 to 128 µM.

In numerous previous studies of drug binding to proteins, the following equation have been applied to quantify the fluorescence quenching:

$$Q = (F_{0} - F)/F_{0}$$

where *F*_0_ and *F* are fluorescence intensities in the absence and presence of NPs, respectively^25^. We assume that the binding of proteins to nanoparticles occurs in equilibrium, and correspondingly, we fit our fluorescence quenching data for *Q* to determine an association constant *K* describing the nanoparticle-protein interaction. The “association constant” *K* is defined to be the reciprocal of the “dissociation constant” *k*_D_.

At low NP concentrations, fluorescence quenching is dominated by diffusive transport, and a non-equilibrium model for the fluorescence quenching is appropriate. The standard model for this regime is attributed to Stern–Volmer^26^ (results section). In particular, the ratio *F*_0_/*F* at low concentrations is predicted to be linear in the concentration of the quenching agent in this theory. The Stern–Volmer equation is traditionally used to quantify fluorescence quenching efficiency by additives at low concentrations that bind or otherwise interact with the fluorescent species^27^. It is also could be used to determine the fluorescence quenching mechanism of the quenchers.

Since a given protein can be expected to have multiple associative interactions with NPs, we can expect the binding equilibrium to exhibit cooperativity, as in the classic example of binding of multiple O_2_ molecules to hemoglobin. Conventionally, this complex phenomenon is taken into account by modeling *Q* through the Hill equation^28^:

$$Q{/}Q_{max} = \left[ {NP} \right]^{n} {/}(k_{D}^{n} + \left[ {NP} \right]^{n} )$$

where *Q*_max_ is the saturation value of *Q*, *k*_D_ is the protein-NP equilibrium constant, and *n* is the Hill coefficient^29^. Although the modeling on which the above equation is based is somewhat idealized, *n* is generally regarded as a measure of association “cooperativity”. For a positively cooperative reaction, *n* > 1, meaning that once one protein molecule is bound to the NP, its affinity for the NP progressively increases in a super linear fashion. For a negatively cooperative reaction, *n* < 1, and the binding strength of the protein to the NP becomes progressively weaker as further proteins adsorb. For a non-cooperative association, where *n* = 1, the affinity of the proteins for NPs does not depend on whether other protein molecules are already bound.

Further analysis was performed to investigate the nanoparticle binding sites on the fibrinogen molecule, the protein with high affinity to the surface of MOFs based on SDS-PAGE analysis, and the thermodynamic properties of the fibrinogen-nanoparticles interactions.

## Results and discussion

### Description of crystal structure

**Figure 1:**
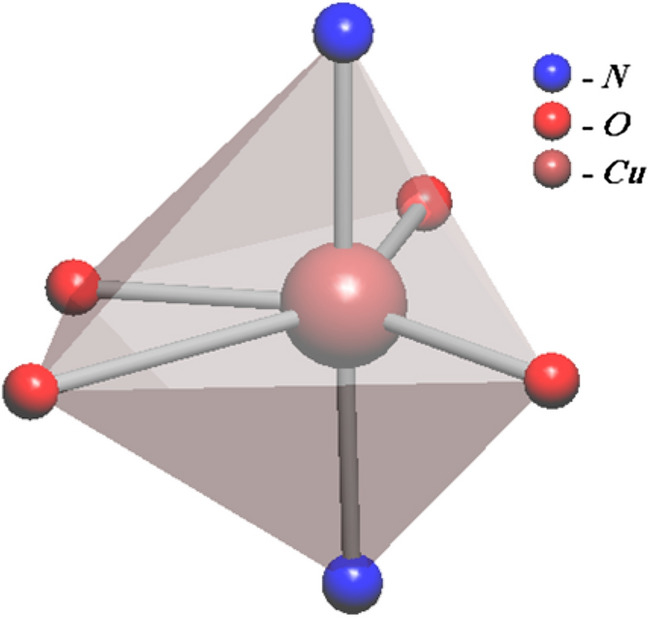
The coordination environment of Cu^II^ cation in compound MOF-1.

The summarized data in Table 1, represents that MOF-1 crystallizes in the orthorhombic system with space group *Pbam*. Each Cu^II^ atom, located in octahedral space, could be octacoordinated owing to surround by four oxygen atoms from 5-aminoisophthalic acid {L^/^} in the equatorial positions and two nitrogen atoms from two bipyridine ligands {L} ligated in axial positions that provide a CuO_4_N_2_ segment (Fig. 1).

The Cu–O bond lengths are located in the range of 1.99 Å to 2.51 Å. The Cu–N distances is 2.01 Å. The L^/^ ligand, as a bridging linker implements a bis(chelate) bridge-monodentate coordination mode (Table 2 and Figure S1).

Using by carboxylate oxygen atoms from L^/^ ligands, Cu and crystallographically equivalent Cu create a [Cu_2_(COO)_4_] dimetallic cluster with a –Cu…Cu separation of 4.35 Å (Figure S2).

The cluster is attached by L^/^ ligands to stretch into a 2D flat layer (Figure S3). The adjacent macrometallocycles associate with together via sharing 5-aip ligands giving birth to the 2D sheet, {Cu(L^/^)}_n_. Then, the neighboring sheets are pillared through the L ligand in longitudinal axes approximately in the distance of 11 Å. Therefore, MOF-1 constructed from the layered motif {Cu(L^/^)}_n_ were connected by the linear bridges into porous 3D networks (Figure S4).

### IR analysis

**Figure 2:**
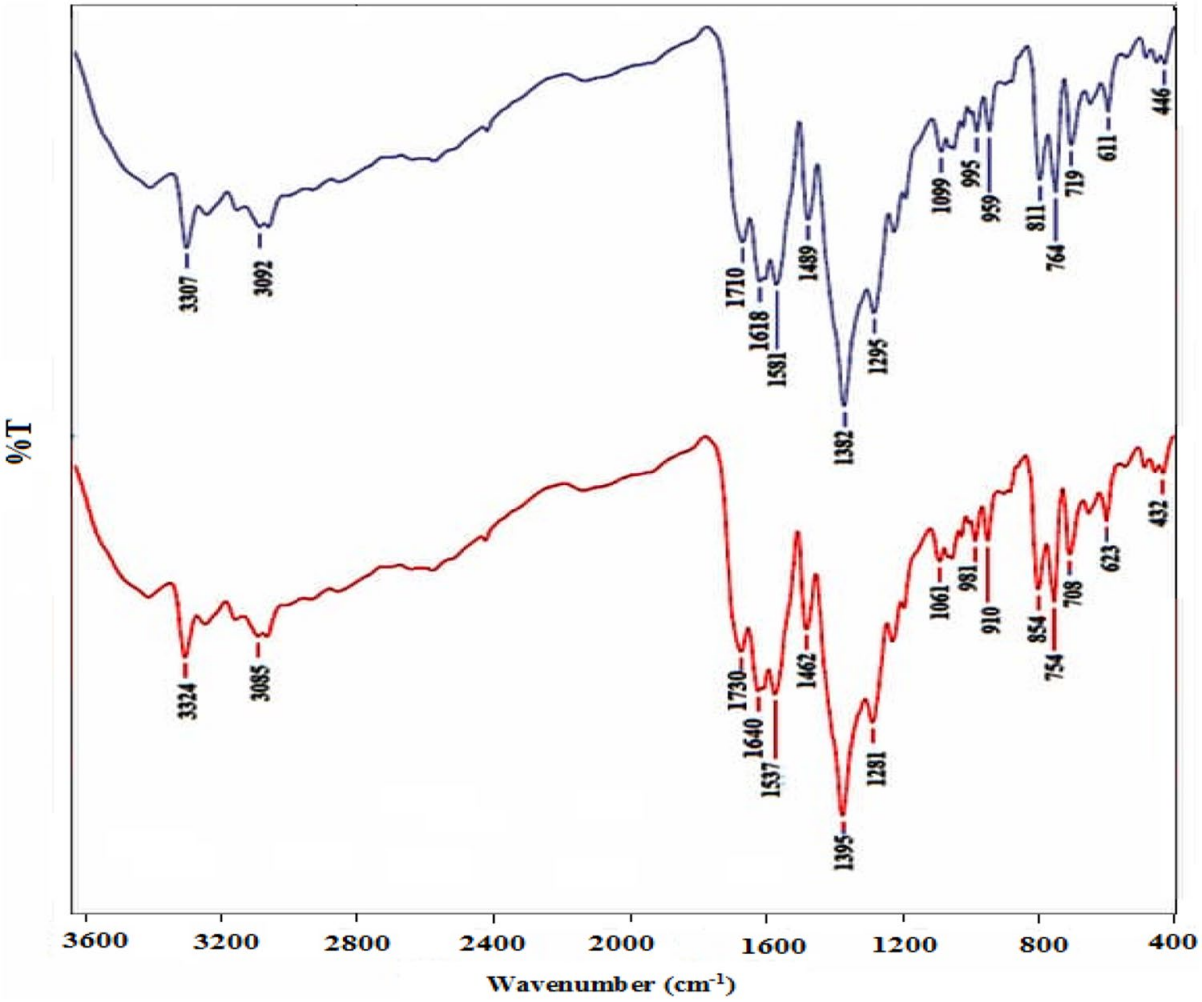
IR spectra of MOF-1 (top: as synthesized microcrystals) and MOF-1s (bottom: sonochemically prepared sample).

The IR spectrum of **1** demonstrated characteristic peaks at around 3,300 cm^−1^ (N–H stretching), 3,000 cm^−1^ (C−H), 1,700–1,750 cm^−1^ (C=O), 1,550–1,500 cm^−1^ (O–C=O), 1,000–1,300 cm^−1^ (C–O). The elemental analysis and IR spectra of different samples of MOF-1s produced by the sonochemical method as well as the bulk material created by the solvothermal method are indistinguishable (Fig. 2). It should be noted that in Table 3, MOF**-**1s is the smallest size of compound MOF-1 which is synthesis via entry five conditions.

**Table 3:** Influence of sonication power, reaction time, concentration of reactants and temperature on the size and morphology of MOF-1s.

Morphology	SEM^d^	Sonication power (W)	Concentration (M)^c^	t (min)^b^	T (°C)^a^	Entry
Cubes (3D)	423.55	0	0.1	60	50	1
Mixed morphology (mostly rods)	114.37	60	0.1	60	50	2
Rods morphology (1D)	191.64	60	0.1	30	50	3
Mixed morphology	345.00	60	0.5	60	50	4
Mixed morphology	46.65	60	0.1	60	70	5 (MOF-1s)^e^

^a^Reaction temperature.

^b^Reaction time.

^c^Concentration of reactants.

^d^Average diameter of particles (nm).

^e^Entry 5 was used to application.

### PXRD and TGA Analysis

**Figure 3:**
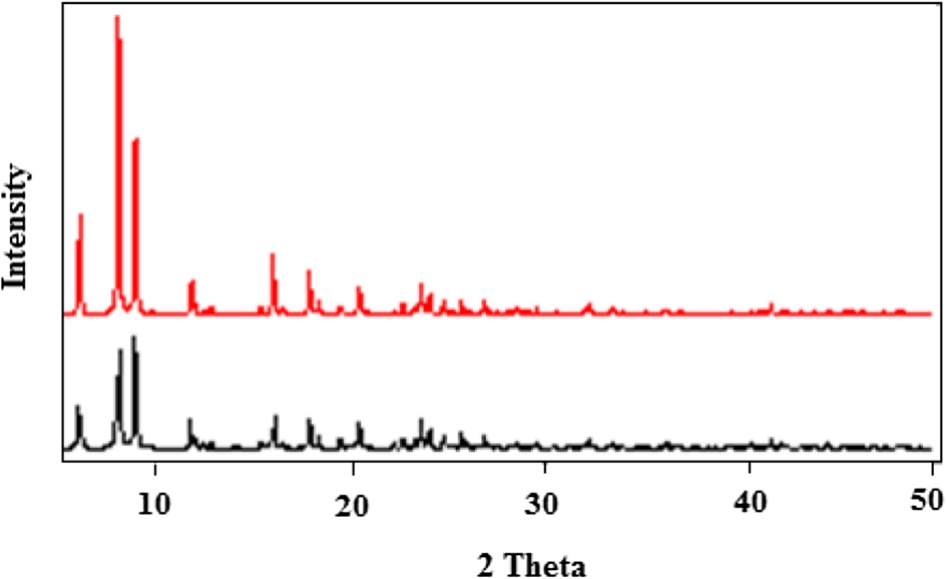
PXRD patterns: simulated from single crystal X-ray data of MOF-1 (top: red line) compound MOF-1s (bottom, black line: sonochemical synthesis).

**Figure 4:**
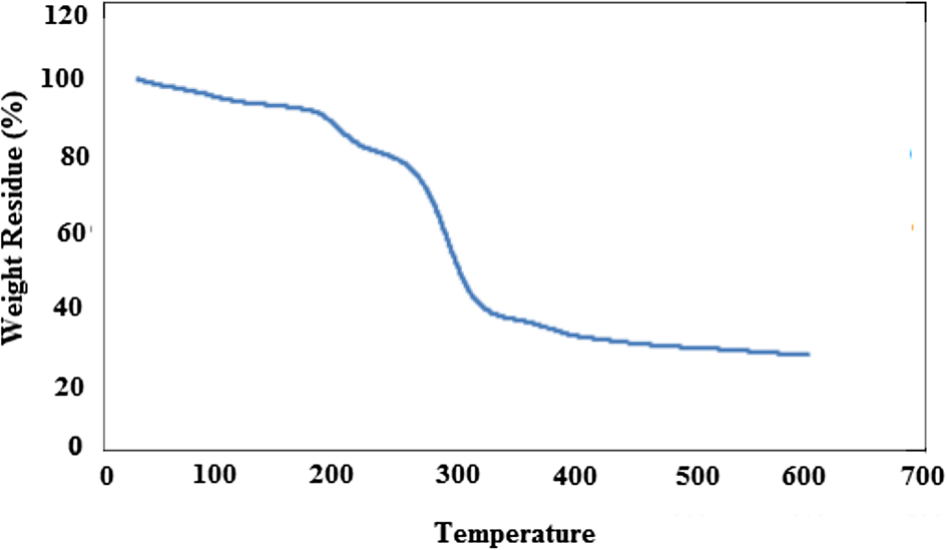
Thermal gravimetric analysis of MOF-1s compound.

As shown in Fig. 3, there is a satisfactory concordance between the PXRD pattern of MOF-1 (simulated from single-crystal X-ray data) and MOF-1s (prepared by the sonochemical process via fifth condition). Indeed, the samples of MOF-1s obtained by the sonochemical process (in a form of micro/nanostructural materials) are identical to single crystals of MOF-1 obtained by a solvothermal method. The wide peaks in the PXRD pattern of MOF-1s could be attributed to sub-micrometer dimensions of the particles. To assess the thermal stability of MOF-1s, thermal gravimetric analysis (TGA) was conducted in the 30–600 °C temperature range under argon flow (Fig. 4). Up to 87.06 °C, the compound was stable, while after that a multistep decomposition was occurred up to 450 °C with a total mass loss of 73.50%.

### Morphology of sonochemically generated samples MOF-1

According to the literature review, sonochemical technique is a promising method to fabricate the coordination polymers, providing good product yields under relatively simple reaction conditions with aim of lower waste production as well as better mass transfer compared to other techniques like layering, solvothermal and diffusion approaches^30,31^.

**Figure 5:**
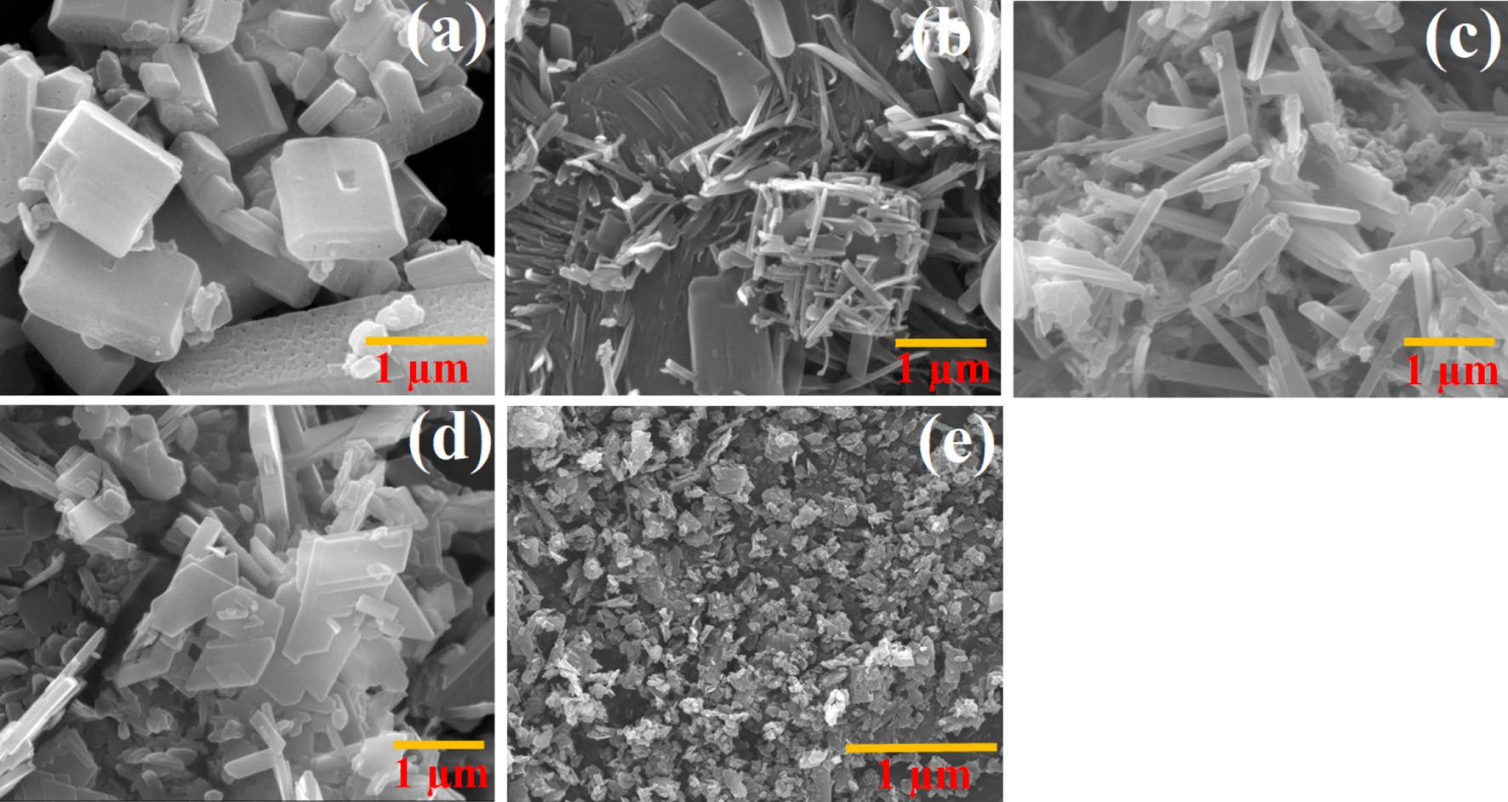
SEM images and the corresponding particle size distribution histogram of micro- and nanosize particles: (**a**) MOF-1 without sonochemical reaction, (**b**) MOF-1 by sonochemical reaction (50 °C, 60 min reaction time, concentration of reactants 0.1 M, 60 W power), (**c**) same as (**b**) but 30 min reaction time, (**d**) same as (**b**) but with concentration of reactants of 0.5 M, (**e**) MOF-1s same as (**b**) but at 70 °C.

SEM images of MOF-1 produced by an ultrasonic generator (60 W) and initial reagent concentrations of [Cu^2+^] = [L] = [L^/^] = 0.1 mol L^−1^ demonstrate a mixed-shape, mainly rod and cube morphologies, accompanied by microscale agglomerates. Various reaction conditions were further tested (Table 1, Fig. 5). As an appropriate result, it was observed that the particle size increases with the increase in the concentration of reactants; however a converse effect was detected after increasing the reaction temperature (Figs. 5b,e). According to the obtained results, under conditions of 60 W ultrasonic irradiation, 60 min, 0.1 M concentration of reagents and temperature 70 °C, a less agglomerated nanostructure of MOF-1s was generated (Table 3, Fig. 5e and Figure S5). Upon ultrasonic treatment the cubes morphology (3D) can be partly converted to small rod morphology (1D), as well.

### Protein corona preparation analysis

#### Preparation and analysis of hard corona on the MOF-1s surface

**Figure 6:**
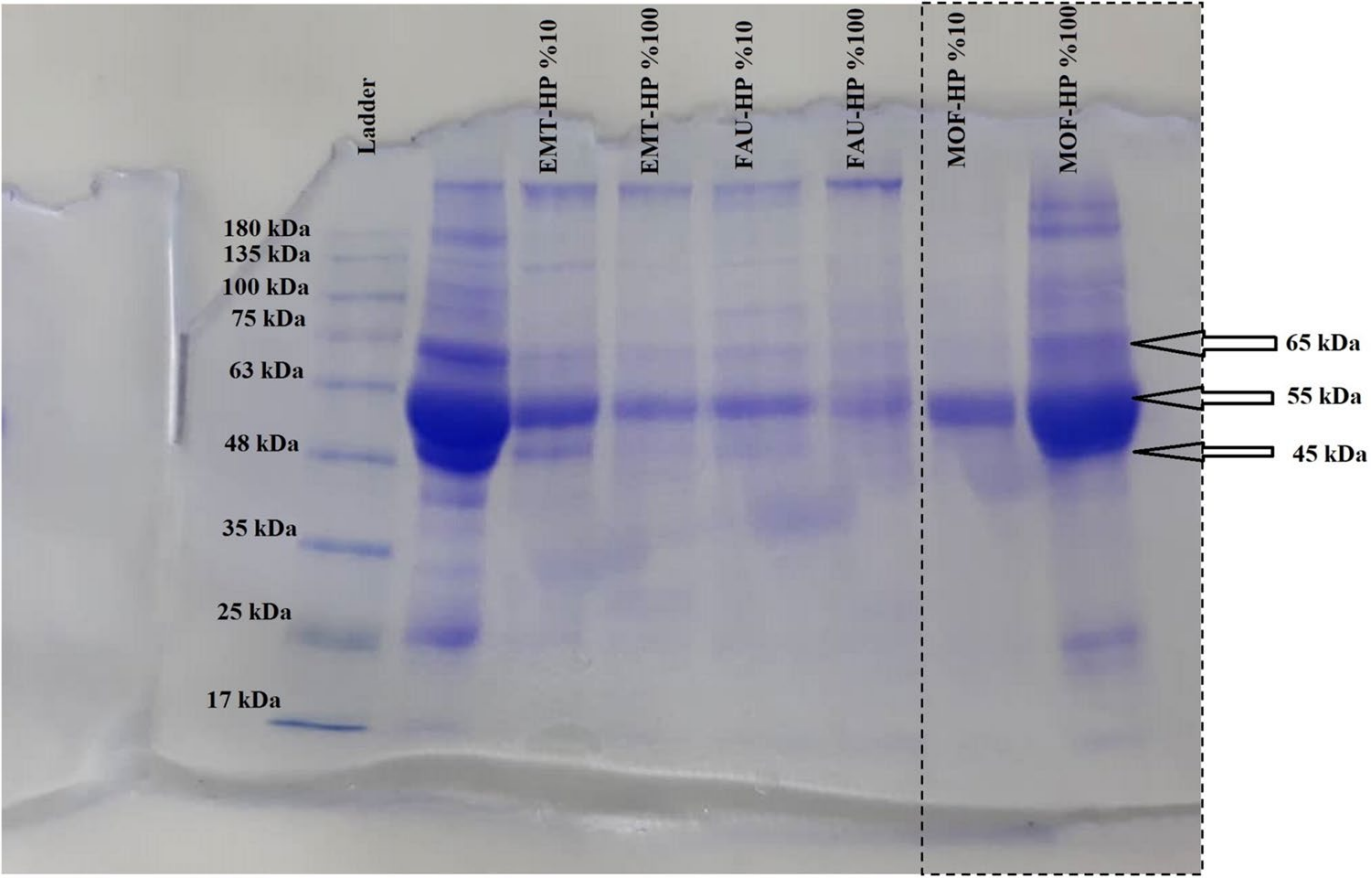
The SDS-PAGE resulted from hard corona of fibrinogen-MOF in different concentrations of human plasma (10 and 100%). Because different nanoparticles apply at the same time on the same gel, the related nanoparticles (MOF) depicted by dashed lines.

When nanoparticles enter a biological environment, various types of molecules such as proteins, lipids, etc*.*, are attached to their surface. Nanoparticles have new nature, which is different from their synthetic identity. As a result, what biological barriers and cells are exposed to these nanoparticles are protein-bound nanoparticles. Therefore, in the present work, the characteristics of these nanoparticles are qualitatively presented using one dimensional SDS-PAGE technique in Fig. 6.

In this study, MOFs coatings were subjected to two different plasma types (10 and 100%). Figure 6 shows the SDS-PAGE corresponding to synthetic MOF in two different concentrations of human plasma (10 and 100%). As mentioned above, SDS-PAGE only qualitatively shows the difference between different concentrations of proteins in various nanoparticles. As it can be seen from Fig. 6, the concentration of adsorbed proteins on the surface of the MOF-1s nanoparticles at higher concentrations is higher than at lower concentrations. For MOF nanoparticles and both plasma concentrations studied, sharp bands in the molecular weights of 45, 55 and 65 kDa were found that coincide with alpha, beta and gamma chains of fibrinogen. Although SDS-PAGE qualitative studies the amount of corona protein, it can be concluded that fibrinogen levels in comparison with other plasma proteins showed higher affinity to MOF nanoparticles.

### Fluorescence quenching studies

#### Interaction of fibrinogen with MOF-1s

**Figure 7:**
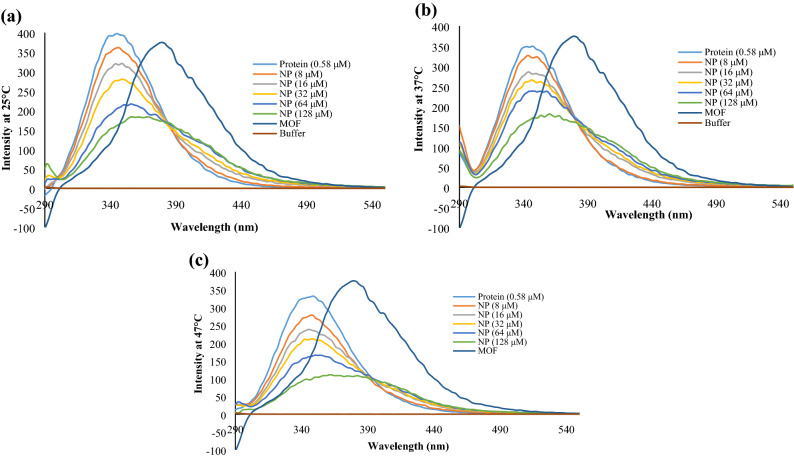
Fluorescence intensity of fibrinogen in presence of different concentrations of MOF-1s.

The fluorescence quenching is a very useful tool that provides much information about interaction, rearrangement, ground-state complex formation, excited-state reactions, energy transfer and collision in molecular levels^32^. In addition, the binding information between small molecules and proteins, such as binding mechanisms, binding sites, binding constants, and intermolecular distance can be revealed by this technique^33^. Moreover, it could provide us with good information about protein-nanoparticle interactions. The fluorescence spectra of fibrinogen in the presence of various concentrations of MOF-1s at 25, 37 and 47 °C are shown in Fig. 7. Fluorescence spectra of buffer was shown as a base line; fibrinogen has maximum fluorescence quenching at about 345 nm that differ from MOF maximum fluorescence quenching (370–380 nm) as a control for all the temperatures.

In all concentrations of MOF-1s particles, the fluorescence peak of fibrinogen was observed about 342.5 nm in case of treated fibrinogen with MOF-1s. In the cases of the gradual decrease in fibrinogen, fluorescence intensity implied that the nanoparticle acts as a quencher, and revealed that the interaction between nanoparticle and fibrinogen occurred. These changes demonstrated the strong interaction between fibrinogen and the nanoparticle, which lead to Tryptophan amino acid microenvironment transition to polar environment and consequent induced conformational changes in fibrinogen structure like a partial unfolding, and solvent exposing of some internal structural binding sites.

#### Quenching behavior of MOF-1s in contact with fibrinogen

In order to determine the molecular quenching mechanism, the fluorescence quenching results were analyzed by Stern–Volmer equation. As we know, there are two quenching mechanisms including static quenching through the formation of a ground-state complex between fluorophore and quencher; and dynamic quenching which depends on diffusive collisions between the excited fluorophore and the quencher ^34,35^.

In dynamic quenching, diffusion has the main role in collisions between fluorophore and quencher. It has also a dependence on temperature so that high temperature causes a faster diffusion, further collision, and a bigger diffusion coefficient. The static quenching, however, has the reversed effect where dissociation of weak bonds in complexes is observed in high temperatures. Determination of quenching rate constants is a good criterion for mechanism detection and judgment between static and dynamic quenching. Thus, the Stern–Volmer equation was used to determine the fluorescence quenching mechanism^36^:

$$F_{0} {/}F = 1 + K_{SV} \left[ Q \right] = 1 + K_{q} \tau \left[ Q \right]$$

**Figure 8:**
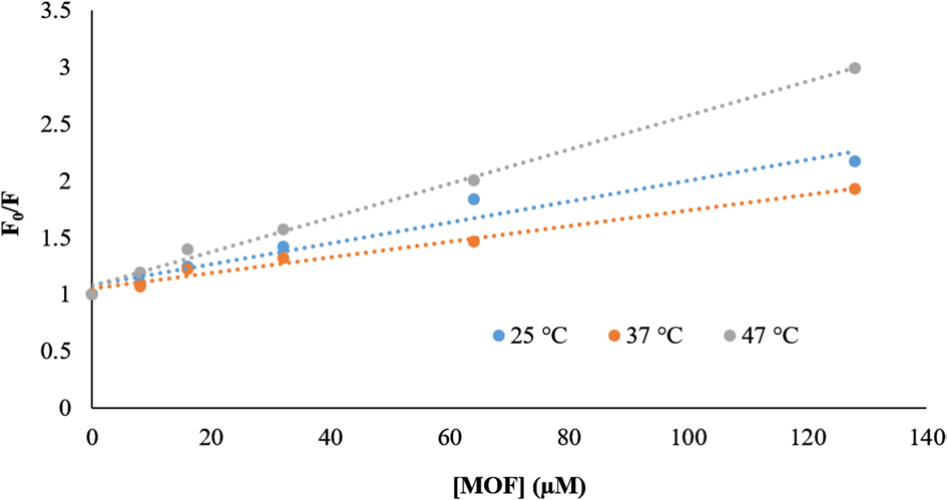
The Stern–Volmer plots of fibrinogen quenching by different concentrations of MOF-1s at different temperatures.

F_0_ and F reveal the fluorescence intensities at steady-state of fibrinogen in the absence and the presence of quencher (MOF). K_SV_ is the Stern–Volmer quenching constant and is gained from linear regression of Stern–Volmer equation; K_q_ is the quenching rate constant of the protein whose maximum value is known to be 2.0 × 10^10^ L mol^−^1s^−1^^37^. [Q] is the concentration of quencher (MOF-1s). τ is the average lifetime of the fluorophore/biomacromolecule in the absence of the quencher. The Stern–Volmer plots of fibrinogen quenching by different concentrations of MOF-1s at different temperatures are displayed in Fig. 8.

The determined amounts of Stern–Volmer quenching constants of MOF-1s are summarized in Table 4.

**Table 4:** The comparison between Stern–Volmer constants of fibrinogen-MOF-1s.

NPs	T (K)	K_sv_ (M^−1^)	K_q_ (M^−1^ S^−1^)	R^2^
MOFs	298.15	9,225.4	92.2 × 10^10^	0.95
	310.15	6,891	68.9 × 10^10^	0.98
	320.15	15,012	15 × 10^10^	0.99

The results in Fig. 8 and Table 2 suggest that the Stern–Volmer plot of MOF-1s is linear. It is noteworthy that, with temperature increasing, the slope of the MOF’s plot and its Ksv increased, and revealed that the MOF-1s fluorescence quenching is followed by dynamic mechanism^38^.

#### Binding sites analysis

The following double–logarithm equation provides more information about binding equilibrium^39^:

$$Log\left( {\left( {F_{0} - F} \right){/}F} \right) = LogK + nLog\left[ Q \right]$$

**Figure 9:**
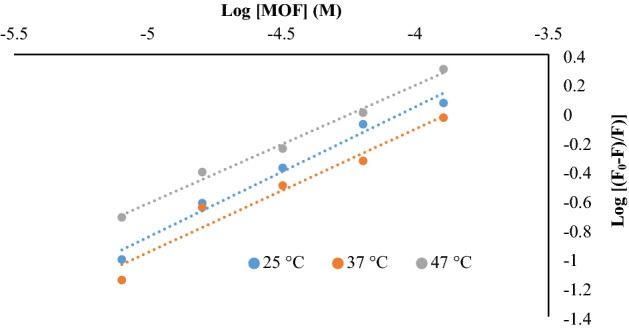
The double-log plot Log ((F_0 _− F)/F) versus Log [MOF] for binding of MOF-1s with fibrinogen in different temperatures.

In this equation, n is the number of binding sites per protein and K is the association constant. The double-logarithm plot for exhibition the binding of MOF-1s with fibrinogen in different temperatures is indicated in Fig. 9. The related results are given in Table 5.

**Table 5:** Binding parameters of MOF-1s interaction with fibrinogen in different temperatures.

NPs	T (K)	K_α_ (M^−1^)	n	R^2^
MOFs	298.15	4,073.8	0.89	0.97
	310.15	1,862.08	0.84	0.95
	320.15	2,691.53	0.81	0.99

According to results, the average number of binding sites per each fibrinogen molecule for MOF-1s nanoparticle is almost independent of the temperature. The number of binding sites of fibrinogen for MOF-1s is about 1^40^.

#### Cooperativity assay

**Figure 10:**
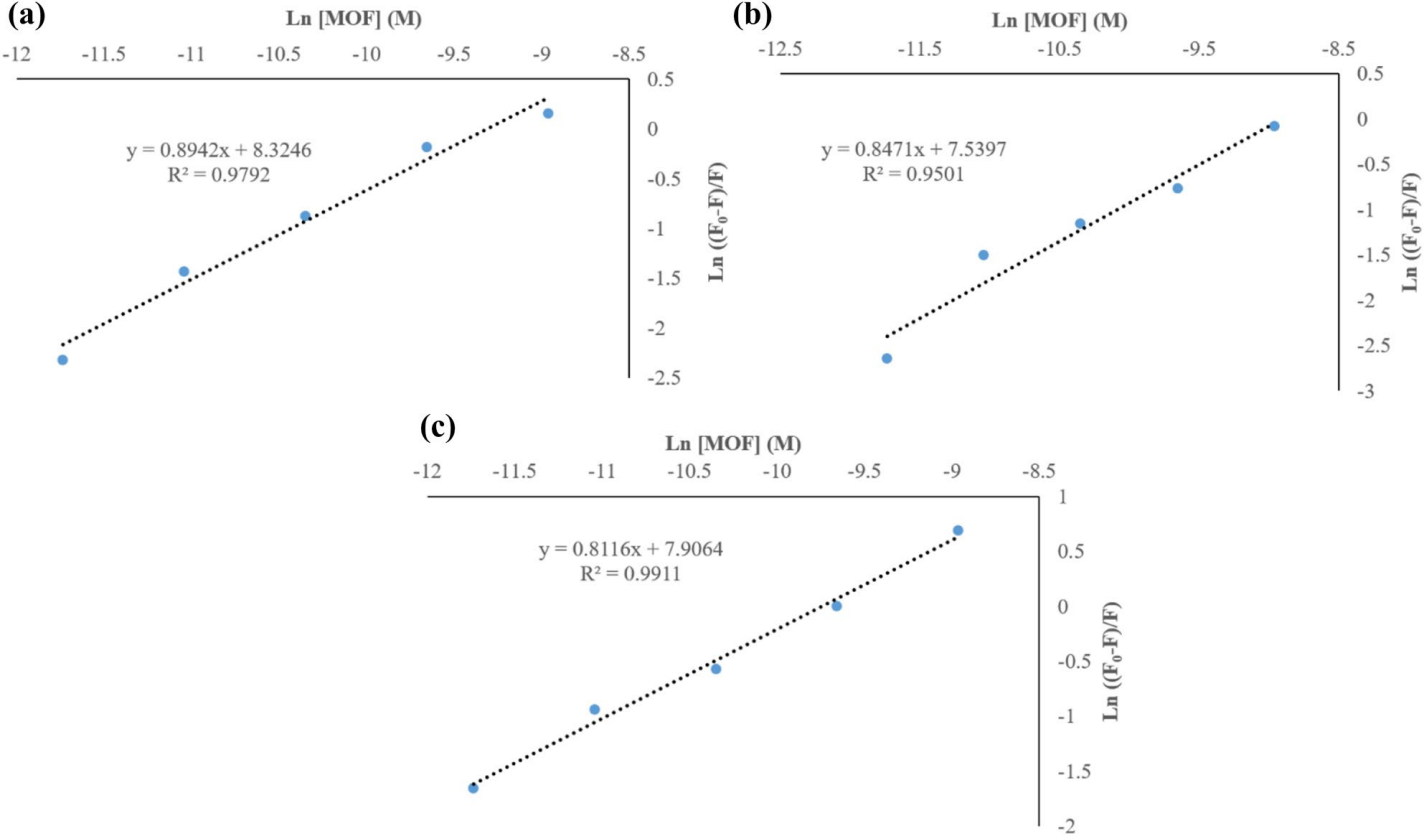
The Hill plot Ln (F_0 _− F)/F)) versus Ln [MOF] for binding of the nanoparticle with fibrinogen at (**A**) room temperature (25 °C), (**B**) 37 °C and (**C**) 47 °C.

To characterize the cooperativity in protein–ligand interaction, and better recognizing about the molecular basis of cooperative binding effects or allosteric effects of MOF-1s in fibrinogen–nanoparticle interactions, the Hill equation was used and Hill coefficient was measured at 25 °C through the relation:

$$Ln\left( {\left( {F_{0} - F} \right){/}F} \right) = nLn\left[ Q \right] - nLnk_{D}$$

where F_0_ and F reveal the fluorescence intensities of fibrinogen in the absence and presence of ligand (MOF-1s), respectively. [Q] is the concentration of ligand (MOF-1s). n and *k*_*D*_ are Hill coefficient and k_dissociation_, respectively. With regard to the Hill equation, in the plot of Ln ((F_0 _− F)/F) versus Ln [Q], n is the slope of the curve (Fig. 10). The Hill plot of fibrinogen in the presence of various concentrations of MOF in 25, 37 and 47 °C are shown in Fig. 10. The Hill coefficient, n, in the fibrinogen-MOF complex at physiological temperature (25 °C) is approximately 1.

#### Thermodynamics analysis

The hydrogen bond, hydrophobic interaction, van der Waals and electrostatic forces are the main interactive forces which involve in molecular interactions between protein and small molecules^41^.

Obtaining the thermodynamic parameters of protein–ligand interaction is the best way for determining the interaction type. The thermodynamic parameters, such as enthalpy $\left( {\Delta H^{0} } \right)$, Gibbs free energy $\left( {\Delta G^{0} } \right)$ and entropy changes $\left( {\Delta S^{0} } \right)$ are the main indices for force determination^42^. The hydrophobic association was defined by $\Delta H^{0}$ > 0 and $\Delta S^{0}$ > 0; Van der Waals forces or hydrogen bond formation by $\Delta H^{0}$ + 0 and $\Delta S^{0}$ < 0; and $\Delta H^{0}$ < 0, and $\Delta S^{0}$ + 0 imply to an electrostatic force. The value of a thermodynamic function in molecular interaction can be estimated from the temperature dependence of quenching constants. Free energy changes of the interaction between fibrinogen and the nanoparticles can be obtained from the following formula for each temperature^43^:

$$\Delta G^{0} = - RTLnK$$

In this equation, K is the association/binding constants for MOF-1s; T is the temperature of experiments and R is the gas constant (8.314 J mol^−1^ K^−1^). By assuming that $\Delta H^{0}$ and $\Delta S^{0}$ values do not vary significantly over the temperature, the ΔG was calculated from Gibbs equation and other parameters can be obtained by plotting the binding and quenching constants according to van’t Hoff equation^42^:

$$LnK = - (\Delta H^{0} {/}RT) + (\Delta S^{0} {/}R)$$

**Figure 11:**
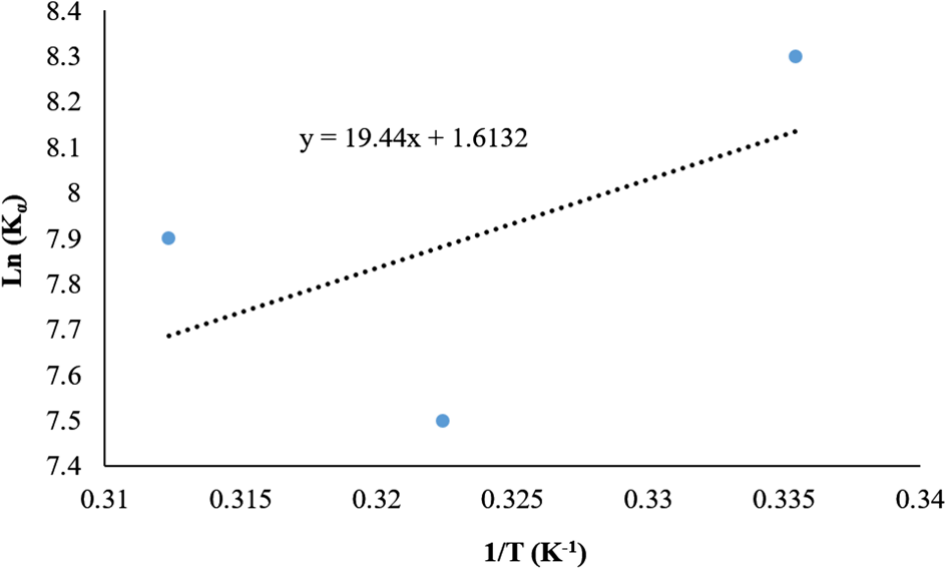
The van’t Hoff plot of MOF-1s in interaction with fibrinogen.

The van’t Hoff plots of fibrinogen- MOF-1s is shown in Fig. 11 and the results are described in Table 6. The negative values of Gibbs free energy for MOF-1s mean that the interaction between MOF-1s with fibrinogen is spontaneous. $\Delta H^{0}$ has negative value ($\Delta H^{0}$ < 0) and $\Delta S^{0}$ has positive value ($\Delta S^{0}$ > 0) for MOF-1s, which indicates that the electrostatic force may play a major role in the interaction process and the reaction was made more favorable by high temperat^44^.

**Table 6:** Thermodynamic parameters of MOF-1s interaction with fibrinogen.

NPs	T (K)	ΔG^0^ (kJ mol^−1^)	ΔH^0^ (kJ mol^−1^)	ΔS^0^ (J mol^−1^ K^−1^)
MOFs	298.15	− 20.5		
	310.15	− 19.3	− 16.1	13.3
	320.15	− 21		

### Cytotoxicity studies and impact of the protein corona on the cytotoxicity of MOF-1s

**Figure 12:**
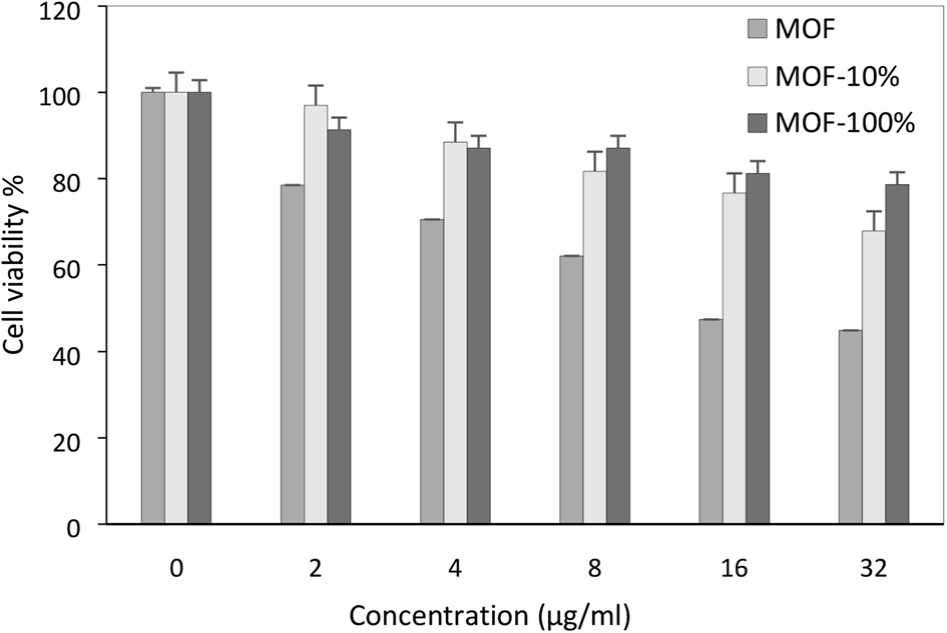
The cytotoxicity of the treated cells with different concentrations of MOF-1s and the exposed MOF with 10 and 100% human plasma. Each point is an average of four replications and the vertical bars represent standard deviations.

The MTT assays were carried out on MCF-7 cells. The toxicity of the treated cells with different concentrations of MOF-1s and the exposed MOF-1s with 10 and 100% human plasma was determined. The result showed that these structures (MOFs) have concentration-dependent toxicity and their toxicity increase at higher concentrations (Fig. 12). On the other hand, the study of the effect of protein corona on the toxicity of MOFs with the plasma proteins showed a reduced toxicity at both concentrations (10 and 100% plasma). High chemical and/or structural versatility (metal, organic bonding, and MOF-1s structure) are factors that influence the toxicity of these nanostructures.

According to previous studies, the exposure of nanoparticles with plasma proteins can mask the properties of them and can be considered as a factor that changes the cytotoxicity of NPs^13,45^. Many toxicity properties of NPs derive from the reactivity of their surface in interfacing cellular membrane and by blocking direct surface contact between NPs and cells can expect these responses to change^13^. However, synthesized MOF-1s have a negatively charged surface and, in complex with the copper ion finds an overall neutral charge that it has been resulted in the increase of protein adsorption on MOF-1s surface and hiding the toxic effects of MOF-1s on exposed cancer cells^46^.

This is likely due to the effect of protein corona in protecting the cells from the direct contact with the reactive surfaces of MOF-1s and it can be a positive point that the protein corona increases the safety of the carriers in other ways. Also, the protein coating on these nanostructures in biological fluids can increase the stability of particles. This is a general model that can be used to prevent the diversion of chemical and physical properties of nanoparticles to study contribute of the protein corona to the toxicity of NP.

## Conclusion

Metal–organic frameworks (MOFs) are a new generation of biomaterials, which can be used in biomedical applications (e.g. diagnostics and therapeutics). In current research, the three-dimensional copper-based metal–organic framework was synthesized and characterized. It was in a good geometry and structural properties that was established by X-ray crystallography and microscopic techniques. The crystal structure of MOF-1 was made up of one 3D Metal organic framework and showed that coordination number for the Cu^+2^ ion is found to be six. The impacts of concentration of reactants, ultrasound power, time of reaction, and temperature on the shape of MOF-1, were investigated and two various morphologies {rods (1D) and small cubes (3D) like} of micro and nanostructures of MOF-1s were obtained. For the first time corona composition of MOF-1s nanostructures was investigated in this study. It was found that fibrinogen, main protein in coagulation cascade, has the highest abundance among the plasma proteins on the surface of MOF-1s. Its interaction with fibrinogen was strong and thermodynamically favorable. Furthermore, cell viability in cancerous cell lines was evaluated by MTT assay at the presence and absence of corona. These structures (MOF-1s) have concentration-dependent toxicity and their toxicity increases at higher concentrations. Evaluation of the protein corona on the toxicity of MOF-1s with the plasma proteins showed a reduced toxicity at both concentrations (10 and 100% plasma). As detailed in the results, this decrease in MOF-1 toxicity could be due to masking the factors affecting the toxicity of these nanostructures such as structural and chemical properties and the lack of direct contact of the nanoparticle surface with the cell surfaces. In brief, based on the obtained data in the current study, the designed MOF can be introduced as a new desirable carrier for biomedical applications such as drug/gen delivery, diagnostics and theranostics after further prerequisite assessments.

## Supplementary information

Supplementary information.
