# Supplementary material for: Human plasma protein corona decreases the toxicity of pillar-layer metal organic framework
Source: Sci Rep. 2020 Sep 3;10:14569. doi: 10.1038/s41598-020-71170-z (PMC7471913; doi:10.1038/s41598-020-71170-z)

**Human plasma protein corona decreases the toxicity of pillar-layer metal organic framework**

Samira Jafari^1^†, Zhila Izadi^1^†, Loghman Alaei^2^, Mehdi Jaymand^3^, Hadi Samadian^3^, Vali ollah Kashani^4^*, Hossein Derakhshankhah^1^*, Payam Hayati^5^*, Foad Noori^6^, Kamran Mansouri^7^, Faezeh Moakedi^8^, Jan Janczak^9^, Mohammad Jaafar Soltanian Fard^10^, Nozar Fayaz bakhsh^10^

†Contributed equally

^1^ Pharmaceutical Sciences Research Center, Health Institute, Kermanshah University of Medical Sciences, Kermanshah, Iran.

^2^Department of Biology and Biotechnology, Faculty of Sciences, University of Kurdistan, Sanandaj, Iran

^3^ Nano Drug Delivery Research Center, Health Technology Institute, Kermanshah University of Medical Sciences, Kermanshah, Iran

^4^ Department of Physical Education, Faculty of Human Sciences, Semnan University, Iran.

^5^ [Persian Gulf Science and Technology Park](http://pgstp.ir), Nano Gostaran Navabegh Fardaye Dashtestan Company, Borazjan, Iran.

^6^ Student’s Research Committee, Faculty of Pharmacy, Kermanshah University of Medical Sciences, Kermanshah 6714415153, Iran.

^c^

^7^ Medical Biology Research Center, Health Technology Institute, Kermanshah University of Medical Sciences, Kermanshah, Iran

^8^ Department of Biochemistry and Molecular Biology, School of Medicine, West Virginia University, USA.

^9^ Institute of Low Temperature and Structure Research Polish Academy of Sciences, P.O. Box1410 Okolna 2 str., 50-950 Wroclaw, Poland.

^10^ Department of chemistry, Faculty of chemical science, Firoozabad Branch, Islamic Azad University, Firoozabad, Fars, Iran, P.O. Box 74715-117

Corresponding authors: Hossein Derakhshankhah*, Payam Hayati*, Vali ollah Kashani *

Email:  [Derakhshankhah.hossein@gmail.com](mailto:Derakhshankhah.hossein@gmail.com) [payamhayati@yahoo.com](mailto:payamhayati@yahoo.com) [vkashani@semnan.ac.ir](mailto:vkashani@semnan.ac.ir)

**Figure. S1** Types of coordination modes of L^/^ in compound **1**.


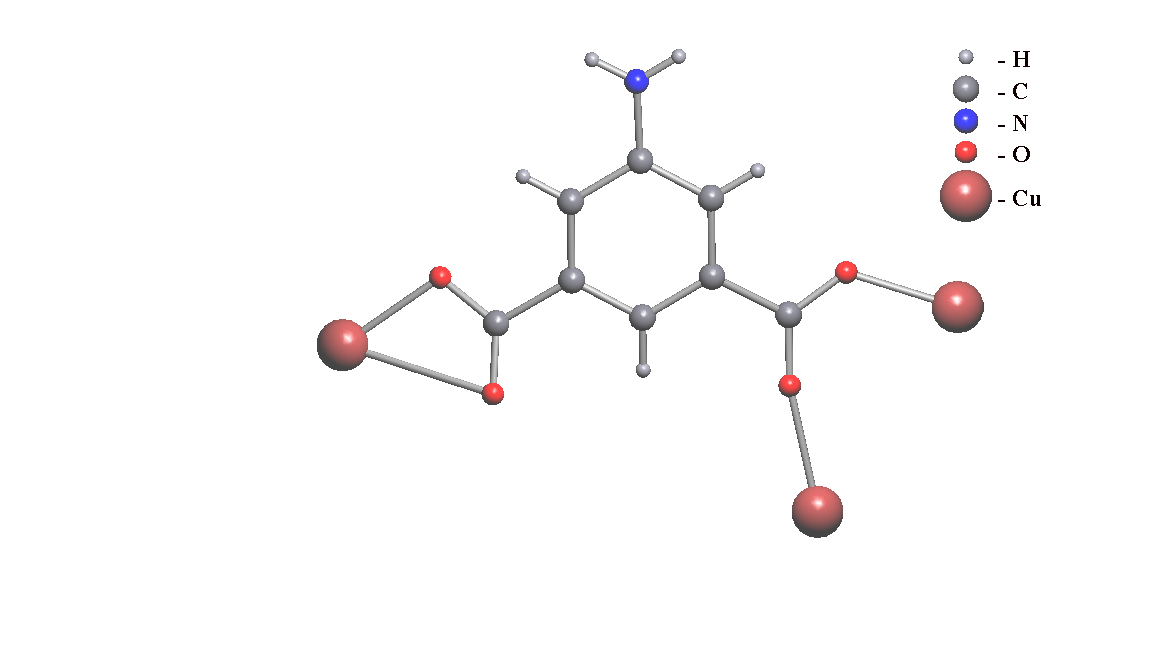


**Figure. S2** Dimetallic cluster of L^/^ ligands in compound **1**.


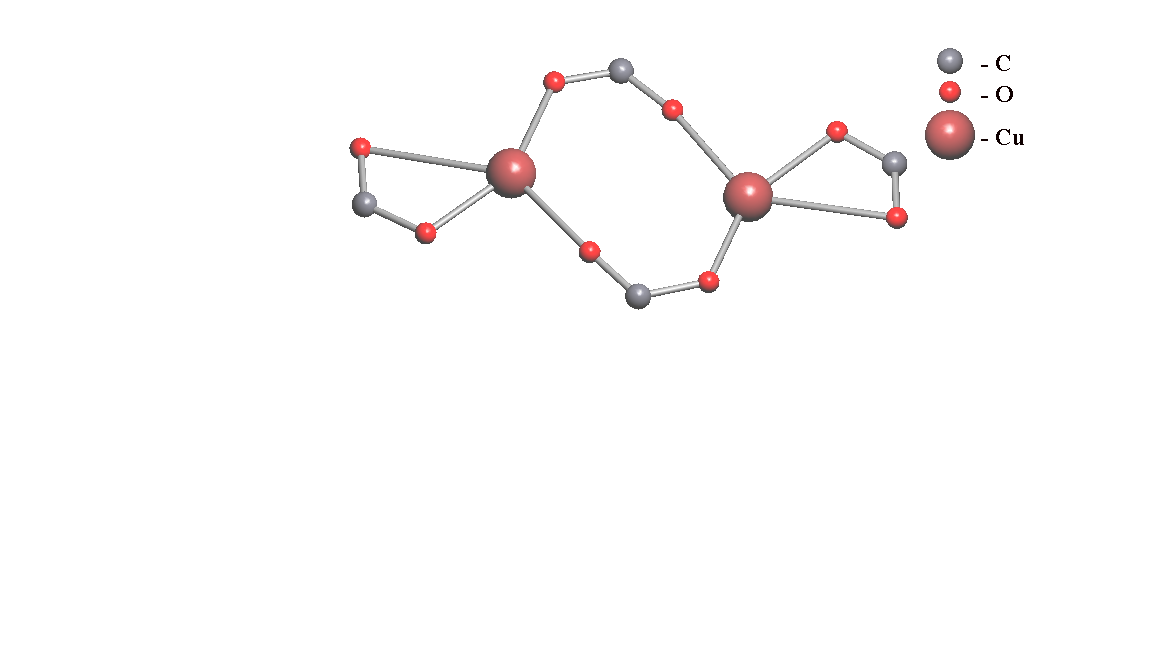


**Figure. S3** 2D layer of L^/^ ligands in compound **1**.


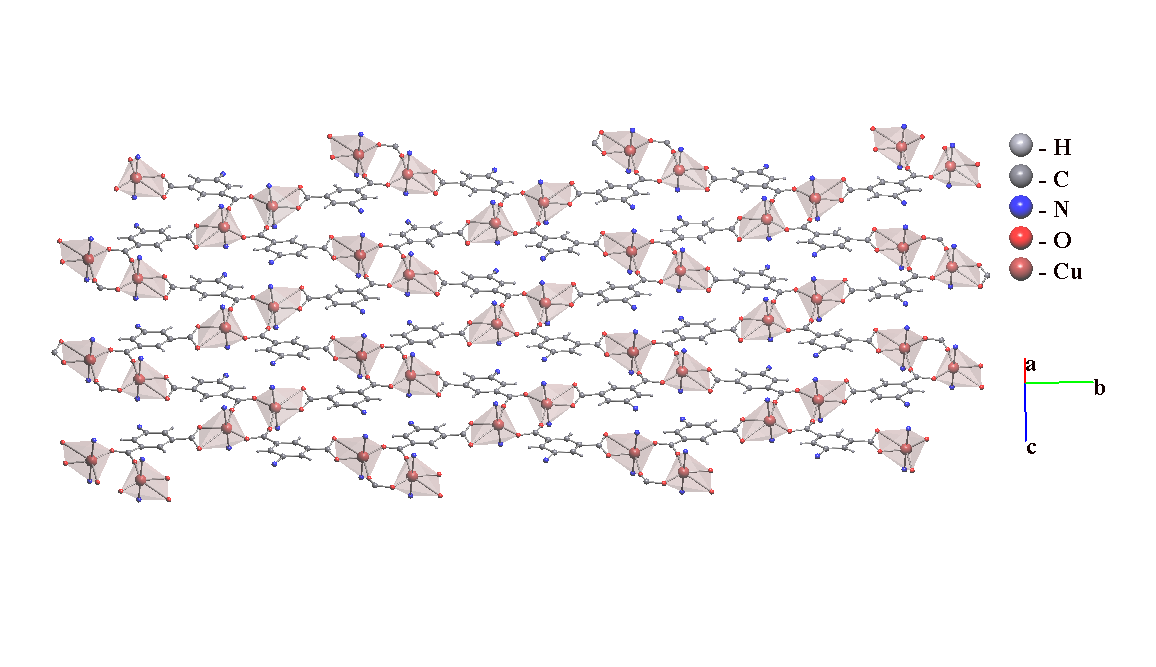


**Figure. S4** Scheme of 3D pillar-layer structure of compound **1**.


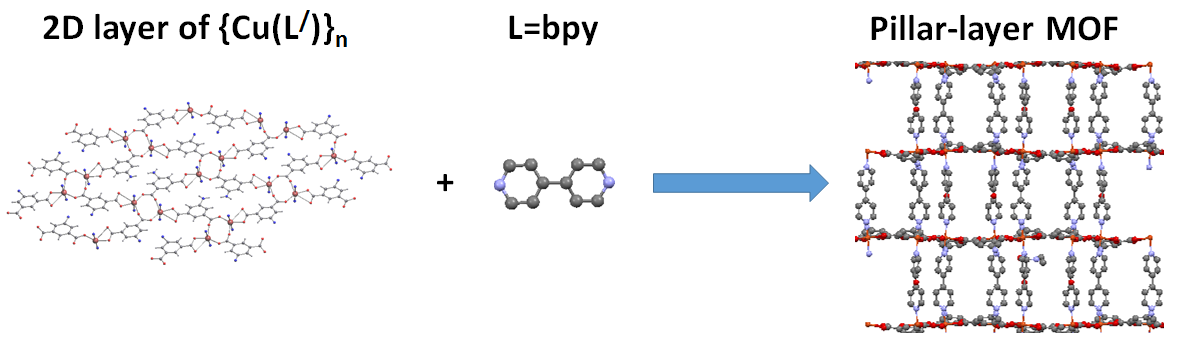


**Figure. S5** SEM photographs and the corresponding particle size distribution histogram

of compound **1** micro and nanostructures.


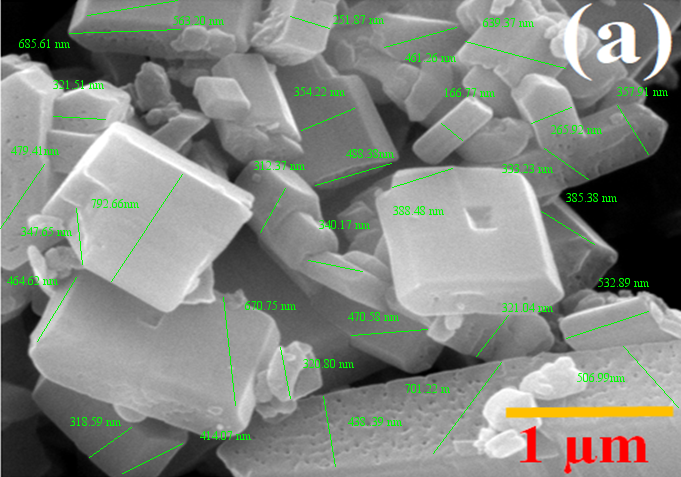


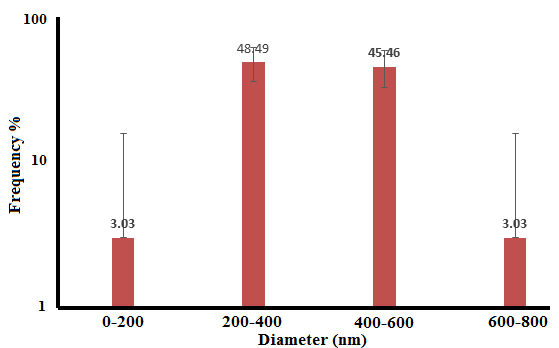


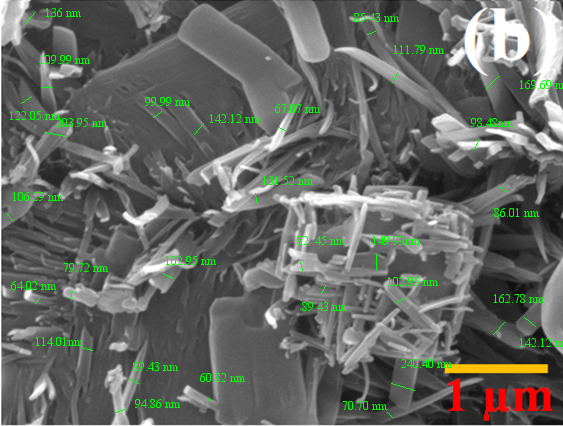

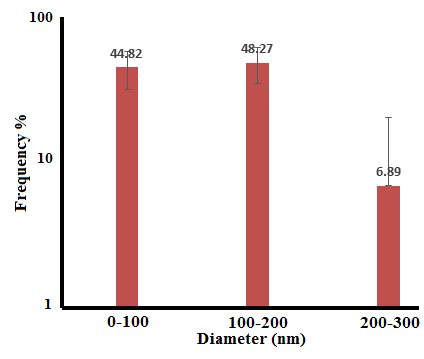


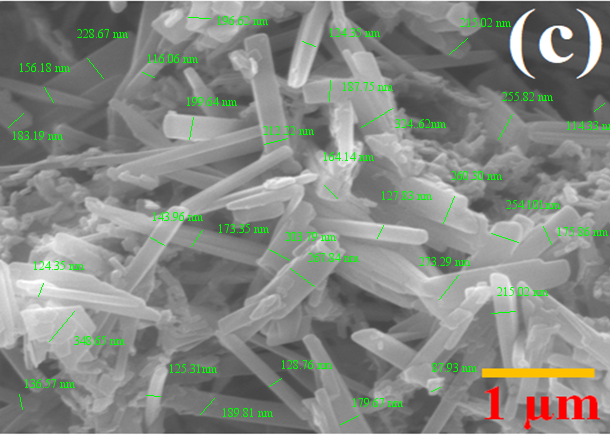


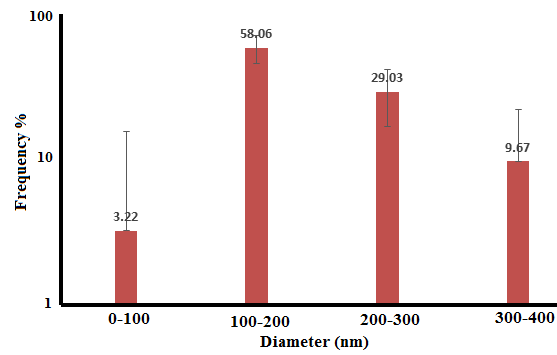


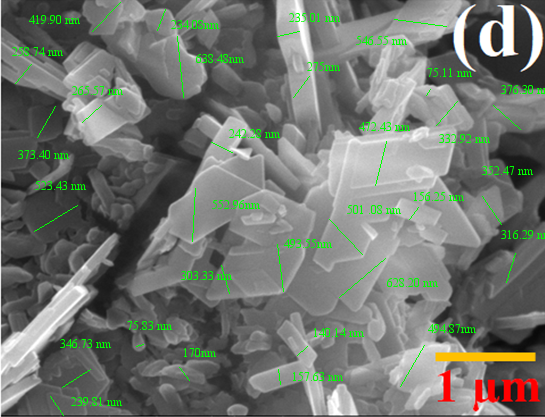


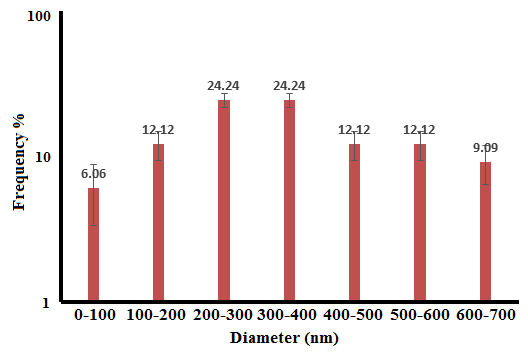


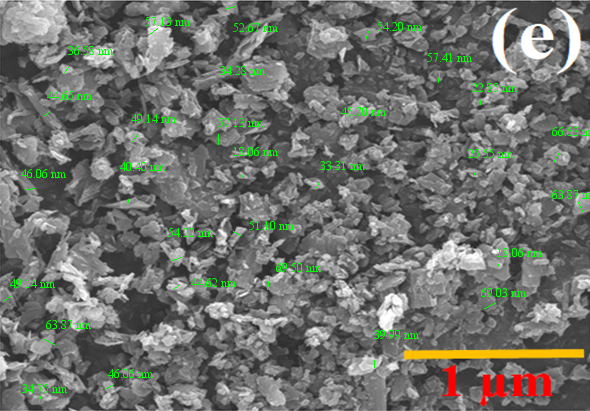


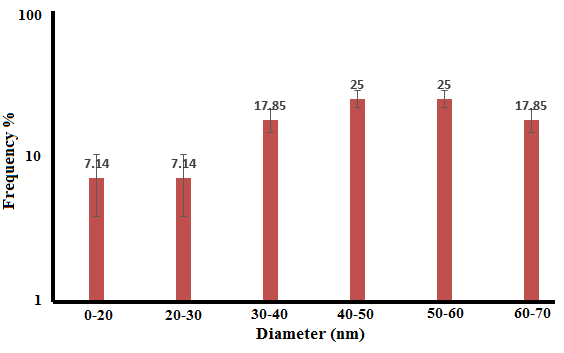

Supplement: Supplementary file 1 — Supplementary information. [file 41598_2020_71170_MOESM1_ESM.docx]
